# Prognostic nomograms for breast cancer with lung metastasis: a SEER-based population study

**DOI:** 10.1186/s12905-023-02848-5

**Published:** 2024-01-03

**Authors:** Yude Xie, Chiseng Lei, Yuhua Ma, Yuan Li, Mei Yang, Yan Zhang, Kin Nam Law, Ningxia Wang, Shaohua Qu

**Affiliations:** Department of Breast Surgery, The First Affiliated Hospital of Jinan University, Jinan University, Guangzhou, China

**Keywords:** Breast cancer, Lung metastases, SEER database, Nomograms, Prognosis

## Abstract

**Background:**

Lung metastasis is a significant adverse predictor of prognosis in patients with breast cancer. Accurate estimation for the prognosis of patients with lung metastasis and population-based validation for the models are lacking. In the present study, we aimed to establish the nomogram to identify prognostic factors correlated with lung metastases and evaluate individualized survival in patients with lung metastasis based on SEER (Surveillance, Epidemiology, and End Results) database.

**Methods:**

We selected 1197 patients diagnosed with breast cancer with lung metastasis (BCLM) from the SEER database and randomly assigned them to the training group (*n* = 837) and the testing group (*n* = 360). Based on univariate and multivariate Cox regression analysis, we evaluated the effects of multiple variables on survival in the training group and constructed a nomogram to predict the 1-, 2-, and 3-year survival probability of patients. The nomogram were verified internally and externally by Concordance index (C-index), Net Reclassification (NRI), Integrated Discrimination Improvement (IDI), Decision Curve Analysis (DCA), and calibration plots.

**Results:**

According to the results of multi-factor Cox regression analysis, age, histopathology, grade, marital status, bone metastasis, brain metastasis, liver metastasis, human epidermal growth factor receptor 2 (HER2), estrogen receptor (ER), progesterone receptor (PR), surgery, neoadjuvant therapy and chemotherapy were considered as independent prognostic factors for patients with BCLM. The C-index in the training group was 0.719 and the testing group was 0.695, respectively. The AUC values of the 1-, 2-, and 3-year prognostic nomogram in the training group were 0.798, 0.790 and 0.793, and the corresponding AUC values in the testing group were 0.765, 0.761 and 0.722. The calculation results of IDI and NRI were shown. The nomograms significantly improved the risk reclassification for 1-, 2-, and 3-year overall mortality prediction compared with the AJCC 7th staging system. According to the calibration plot, nomograms showed good consistency between predicted and actual overall survival (OS) values for the patients with BCLM. DCA showed that nomograms had better net benefits at different threshold probabilities at different time points compared with the AJCC 7th staging system.

**Conclusions:**

Nomograms that predicted 1-, 2-, and 3-year OS for patients with BCLM were successfully constructed and validated to help physicians in evaluating the high risk of mortality in breast cancer patients.

## Introduction

With the increasing incidence and mortality, breast cancer has become the most commonly diagnosed cancer and the leading cause of death in women worldwide. In 2022, about 339,250 cases of breast cancer were diagnosed, which caused 43,250 deaths in the US [1]. Nearly 90% of breast cancer-related deaths are caused by complications of metastasis [2]. Breast cancer is apt to metastasize to bone, lung, liver, and brain preferentially. Among the common metastatic sites, bone metastasis accounts for 30–60%, brain metastasis accounts for 4–10%, liver metastasis accounts for 15–32% and lung metastasis accounts for 21–32% [3].

Lung metastasis, as one of the most common sites of distant metastasis [3], is of particular concern as it is associated with significant patient morbidity and a mortality rate of 60–70% [4]. Lung metastasis has a tendency to occur within 5 years from initial breast cancer diagnosis and causes pulmonary dysfunction leading to symptoms such as cough, chest pain, dyspnea, hemoptysis, and eventual death. The prognosis of patients with lung metastasis is extremely poor, and the median survival time is only 25 months [4]. Therefore, exploring an efficient method to predict the prognosis in patients with lung metastases is extremely important, as this population is growing and has historically been excluded from large clinical trials.

American Joint Committee on Cancer (AJCC) is internationally used as the staging criteria for breast cancer to evaluate the prognosis of patients. However, AJCC staging is categorized only by tumor size and extent (T), lymph node involvement (N), and distant metastasis (M), and patients with metastasis are all categorized as stage IV, which is defined as any histologically proven metastases in distant organs. The prognosis of patients with breast cancer lung metastasis is multifactorial [5]. Accumulating evidence suggests that molecular subtypes as well as other biological factors were correlated with the prognosis of patients with metastases [6–8]. Thus, AJCC may not be adequate for clinicians to evaluate prognosis because of the complexity of metastasis. Accurate estimation for the prognosis of patients with lung metastasis and population-based validation for these models are warranted.

The nomogram is a reliable tool to accurately predict individual prognosis for cancer. Up till the present moment, there has been a variety of prognostic nomograms for patients with metastases, such as nomograms for breast cancer survival with brain metastases [6], bone metastases [7], and liver metastases [8]. These prognostic models are widely used in clinical practice. However, the survival prognostic nomograms of patients with breast cancer with lung metastasis have not been adequately studied, which may be due to several factors such as limited patient follow-up.

In this study, we aimed to construct nomograms using data from the SEER (Surveillance, Epidemiology, and End Results) population-based database to predict the survival of patients with lung metastases and the associated risk factors. Firstly, we used statistical methods to describe sociodemographic and clinicopathological parameters in patients with lung metastases. Secondly, univariate and multivariate Cox regression analysis were used, variable factors related to the prognosis of lung metastases were obtained and survival predictive nomogram were constructed. Our study will help clinicians better understand the survival and risk factors of lung metastases.

## Materials and methods

### Patients

With the help of SEER*Stat software (version 8.4.0.1, National Cancer Institute, Bethesda, MD), we collected detailed data of patients diagnosed with breast cancer (site recode International Classification of Disease for Oncology, site recode ICD-0–3/WHO 2008 of “Breast”) from the SEER program of the National Cancer Institute [9]. The SEER database is publicly accessible (https://seer.cancer.gov/data/access.html). We have received the permission from the SEER registry to access the data (authorization number: 15348-Nov2021). The SEER program provides data on cancer incidence, prevalence, and mortality in the United States, and covers 28% of the US population from 18 cancer registries [10, 11]. The SEER database contains detailed information on patients diagnosed with breast cancer from 2000 to 2018 (2004 AJCC 6th and 2010 AJCC 7th). Molecular subtypes are important factors influencing the prognosis of breast cancer patients. Since the SEER database began collecting information on the molecular subtypes and sites of distant metastasis in 2010, and considering that the data about treatment after 2015 remain incomplete but is essential for the establishment of our nomogram, we therefore extracted breast cancer patients with lung metastases at the time of initial diagnosis from 2010 to 2015 for reasons of data completeness and adequate follow-up time. The patients who fulfilled the following criteria were included: 1) female; 2) age ≥ 20 years; 3) breast cancer is the primary tumor and the only malignant tumor in the patient’s lifetime; 4) lung metastasis; 5) survival time and cause of death (COD) are known. Patients with insufficient or unknown data were excluded.

### Variables

We retrieved demographic, clinicopathological, and therapeutic variables from the SEER database, including age, race, marital status, years of diagnosis, laterality, primary tumor location, tumor size, histopathology, grade, tumor stage (T stage), nodal stage (N stage), metastatic stage (M stage), clinical stage (TNM stage), HER2, ER, and PR stage, to identify the risk factors of breast cancer. In addition, sites of distant metastases including brain, liver, and bone, and therapy methods including neoadjuvant therapy, surgery, chemotherapy, and radiotherapy were added to identify prognostic factors associated with breast cancer with lung metastasis. The 7th edition of the AJCC TNM staging system was applied to the patients in this study. According to World Health Organization [WHO] classification scheme, we used ICD-0–3 code to divide the histological types of breast cancer patients into three groups: ductal carcinoma (ICD-0 codes 8500), lobular carcinoma (ICD-0 codes 8520) and others (carcinoma not otherwise specified [NOS]).

### Construction of the nomogram

After excluding some patients with blank data and follow-up loss, eligible patients were randomly divided into a training group and a testing group with a ratio of 7:3 by R. In this study, data from the training group was used to analyze prognosis and construct the nomogram, whereas data from the testing group was used to verify the prediction model. Variables in this study included age (20–34, 35–59, 60–84,or 85 and above), race (White, Black, or others), marital status (married, unmarried, or others), years of diagnosis (2010–2011, 2012–2013, or 2014–2015), laterality (left or right), primary tumor location (nipple C50.0, central portion of breast C501, upper inner quadrant of breast C502, lower inner quadrant of breast C503, upper outer quadrant of breast C504, lower outer quadrant of breast C505, axillary tail of breast C506, overlapping lesion of breast C508, or breast NOS C509), tumor size (≤ 2 cm, 2.1–5.0 cm, 5.1–10 cm, or > 10 cm), histopathology (ductal carcinoma, lobular carcinoma, others), grade (I ~ II, III, or unknown), T stage (T1, T2, T3, or T4), N stage (N0, N1, N2, or N3), HER-2 (positive, negative, or unknown), ER (positive or negative), PR (positive or negative), bone metastasis (yes, no, or unknown), brain metastasis (yes, no, or unknown), liver metastasis (yes, no, or unknown), surgery (yes or no), neoadjuvant therapy (yes or no), chemotherapy (yes or no), radiotherapy (yes or no). Considering that patients with BCLM were in stage IV, variables of the clinical stage and M stage were excluded.

### Validation of the nomogram

The identification performances of the nomogram were evaluated quantitatively by the Harrell concordance index (C-index) and the area under curve (AUC) of the receiver operating characteristic (ROC) curve. The value of C-index and AUC ranges from 0.5 to 1, with 1 indicating perfect discrimination and 0.5 indicating no discrimination [12]. The net reclassification improvement (NRI) and integrated discrimination improvement (IDI) were performed to evaluate the overall improvement of the nomogram over the TNM staging system for predicting OS in patients. NRI refers to the difference in the proportion of patients with a higher probability of events being correctly assigned and a lower probability of events being correctly assigned in the updated model compared to the original model [13]. NRI, which is based on reclassification tables, has more advantages in evaluating the relative quality of two models at a certain point compared with the AUC value. IDI can be used to evaluate the overall improvement of the model and to evaluate the degree of average sensitivity of the new model over the old model without reducing the average specificity [14]. The nomogram calibration was investigated from the graphical representations of the consistency of the predicted probabilities and the observed outcomes based on 1000 bootstrap resamples [15]. Finally, the decision curve analysis (DCA) was performed to validate the clinical efficacy of the nomogram and the TNM staging system of the older model, which is a method for assessing whether the clinical usefulness of prediction models increased the net benefits when realistic threshold probabilities were considered [16, 17]. These data were analyzed by R version 3.5.1 (http://www.r-project.org/).

### Statistical analysis

The primary endpoint of this study was OS, which is defined as the time interval between diagnosis of breast cancer and death occurring as a result of all causes (including breast cancer) or the last follow-up. We used Kaplan–Meier methods to compute the survival estimates and generate survival curves. Univariate and multivariate Cox regression analyses were conducted to identify the significant prognostic factors of OS using the backward stepwise method. Hazard ratios (HRs) with 95% confidence intervals (CIs) were calculated. A *p*-value < 0.05 was determined as statistically significant. All *p* values were two-tailed. According to the results of the multi-factor analysis, we selected the variables which *p* values < 0.05 to construct a 1-, 2-, and 3-year survival predictive nomogram for patients with BCLM.

## Result

### Patients’ characteristics

In the study, a total of 1,197 patients with BCLM were selected from the SEER database, of which 837 patients were divided into the training group and 360 patients into the testing group. Throughout the study, the overall follow-up ranged from 0 to 119 months, with a median follow-up of 25 months. The follow-up times ranged from 0 to 118 months in the training group and 0 to 119 months in the testing group, with a median follow-up time of 24 and 25.5 months, respectively. There were 810 patients died at the end of follow-up, including 244 (67.8%) patients in the testing group and 566 (67.6%) patients in the training group.

The baseline characteristics of the patients in the training group and testing group were shown in Table 1. The baseline characteristics showed a higher proportion of patients with invasive ductal carcinoma (78.6% & 80.8%), ER positive (70.3% & 71.3%), and HER2 negative (70.0% & 68.9%) subtypes in both cohorts. Patients aged 60–84 (49.6%) and 35–59 (42.1%) accounted for the majority of the population. From the perspective of pathological grading, there were 472 patients (39.4%) in grade I ~ II subgroup and 575 patients (48.0%) in grade III subgroup. In addition, 166 patients (13.9%) underwent neoadjuvant chemotherapy, 716 patients (59.8%) received chemotherapy and 391 patients (32.7%) underwent radiotherapy.

**Table 1:** Patient characteristics of the training group and the testing group (*n* = 1197)

	**Overal** **(*n* = 1197)**	**Testing group** **(*n* = 360)**	**Training group** **(*n* = 837)**	***p***
**Characteristics**	**Number of patients (%)**			
Age (%)				0.41
20–35	32 (2.7)	6 (1.7)	26 (3.1)	
35–59	504 (42.1)	156 (43.3)	348 (41.6)	
60–84	594 (49.6)	181 (50.3)	413 (49.3)	
85 +	67 (5.6)	17 (4.7)	50 (6.0)	
Tumor size (%)				0.09
≤ 2 cm	155 (12.9)	44 (12.2)	111 (13.3)	
> 10 cm	240 (20.1)	61 (16.9)	179 (21.4)	
2.1-5 cm	448 (37.4)	153 (42.5)	295 (35.2)	
5.1-10 cm	354 (29.6)	102 (28.3)	252 (30.1)	
Race (%)				0.255
Black	849 (70.9)	257 (71.4)	592 (70.7)	
Other	139 (11.6)	48 (13.3)	91 (10.9)	
White	209 (17.5)	55 (15.3)	154 (18.4)	
Year of diagnosis (%)				0.894
2010–2011	343 (28.7)	100 (27.8)	243 (29.0)	
2012–2013	421 (35.2)	127 (35.3)	294 (35.1)	
2014–2015	433 (36.2)	133 (36.9)	300 (35.8)	
Laterality (%)				0.329
Left	631 (52.7)	198 (55.0)	433 (51.7)	
Right	566 (47.3)	162 (45.0)	404 (48.3)	
Primary site (%)				0.801
Nipple	5 (0.4)	0 (0.0)	5 (0.6)	
Central portion	63 (5.3)	18 (5.0)	45 (5.4)	
Upper inner quadrant	92 (7.7)	27 (7.5)	65 (7.8)	
Lower inner quadrant	36 (3.0)	11 (3.1)	25 (3.0)	
Upper outer quadrant	284 (23.7)	85 (23.6)	199 (23.8)	
Lower outer quadrant	74 (6.2)	20 (5.6)	54 (6.5)	
Axillary tail	7 (0.6)	3 (0.8)	4 (0.5)	
Overlapping lesion	248 (20.7)	70 (19.4)	178 (21.3)	
Breast NOS	388 (32.4)	126 (35.0)	262 (31.3)	
Histopathology (%)				0.668
Ductal	959 (80.1)	283 (78.6)	676 (80.8)	
Lobular	38 (3.2)	13 (3.6)	25 (3.0)	
Other	200 (16.7)	64 (17.8)	136 (16.2)	
Grade (%)				0.065
I ~ II	472 (39.4)	141 (39.2)	331 (39.5)	
III	575 (48.0)	162 (45.0)	413 (49.3)	
Unknown	150 (12.5)	57 (15.8)	93 (11.1)	
T stage (%)				0.059
T1	133 (11.1)	38 (10.6)	95 (11.4)	
T2	342 (28.6)	121 (33.6)	221 (26.4)	
T3	203 (17.0)	51 (14.2)	152 (18.2)	
T4	519 (43.4)	150 (41.7)	369 (44.1)	
N stage (%)				0.158
N0	270 (22.6)	79 (21.9)	191 (22.8)	
N1	575 (48.0)	164 (45.6)	411 (49.1)	
N2	162 (13.5)	61 (16.9)	101 (12.1)	
N3	190 (15.9)	56 (15.6)	134 (16.0)	
Marital status (%)				0.705
Married	479 (40.0)	138 (38.3)	341 (40.7)	
Other	219 (18.3)	66 (18.3)	153 (18.3)	
Unmarried	499 (41.7)	156 (43.3)	343 (41.0)	
Bone metastasis (%)				0.769
No	535 (44.7)	166 (46.1)	369 (44.1)	
Unknown	12 (1.0)	3 (0.8)	9 (1.1)	
Yes	650 (54.3)	191 (53.1)	459 (54.8)	
Brain metastasis (%)				0.189
No	1036 (86.5)	317 (88.1)	719 (85.9)	
Unknown	32 (2.7)	5 (1.4)	27 (3.2)	
Yes	129 (10.8)	38 (10.6)	91 (10.9)	
Liver metastasis (%)				0.821
No	828 (69.2)	253 (70.3)	575 (68.7)	
Unknown	23 (1.9)	6 (1.7)	17 (2.0)	
Yes	346 (28.9)	101 (28.1)	245 (29.3)	
HER2 (%)				0.819
Negative	829 (69.3)	252 (70.0)	577 (68.9)	
Positive	322 (26.9)	93 (25.8)	229 (27.4)	
Unknown	46 (3.8)	15 (4.2)	31 (3.7)	
ER (%)				0.766
Negative	347 (29.0)	107 (29.7)	240 (28.7)	
Positive	850 (71.0)	253 (70.3)	597 (71.3)	
PR (%)				0.401
Negative	525 (43.9)	165 (45.8)	360 (43.0)	
Positive	672 (56.1)	195 (54.2)	477 (57.0)	
Surgery (%)				0.506
No	857(71.6)	263 (73.1)	594 (71.0)	
Yes	340 (28.4)	97 (26.9)	243 (29.0)	
Neoadjuvant therapy (%)				0.241
No	1031 (86.1)	317 (88.1)	714 (85.3)	
Yes	166 (13.9)	43 (11.9)	123 (14.7)	
Chemotherapy (%)				0.983
No	481 (40.2)	144 (40.0)	337 (40.3)	
Yes	716 (59.8)	216 (60.0)	500 (59.7)	
Radiotherapy (%)				0.903
No/refuse/unknown	806 (67.3)	241 (66.9)	565 (67.5)	
Yes	391 (32.7)	119 (33.1)	272 (32.5)	
Status (%)				1
Alive	387 (32.3)	116 (32.2)	271 (32.4)	
Dead	810 (67.7)	244 (67.8)	566 (67.6)	

*Ductal* invasive ductal carcinoma, *Lobular* invasive lobular carcinoma

### Survival analysis and nomogram development

**Fig. 1:**
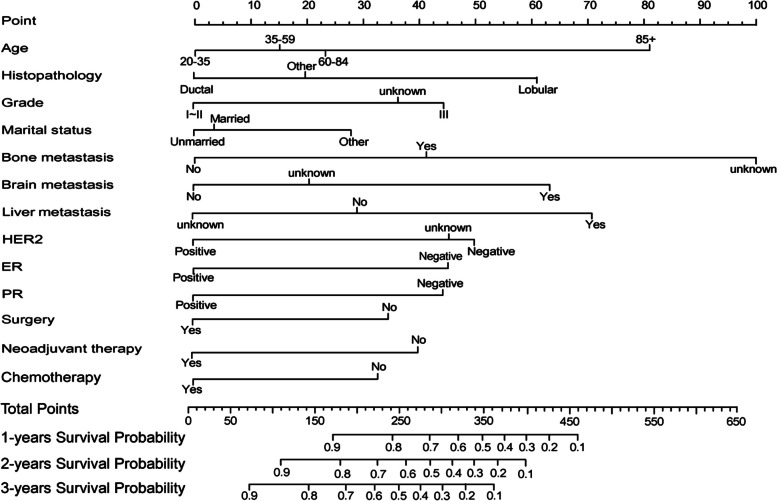
The nomogram for predicting 1-, 2-, and 3-year overall survival of patients with BCLM. Ductal = invasive ductal carcinoma (IDC); Lobular = invasive lobular carcinoma (ILC)

The univariate and multivariate analysis results of the training group were shown in Table 2. Multivariate analyses demonstrated 13 key predictors for OS including age, histopathology, grade, marital status, bone metastasis, brain metastasis, liver metastasis, HER2, ER, PR, surgery, neoadjuvant therapy, and chemotherapy, which have statistical significance (*p* < 0.05) (Table 2). The important factors related to OS were used to construct the nomogram to predict 1-, 2-, and 3-year OS in the training group (Fig. 1). By adding the scores associated with each factor, the OS of the patients with LM could be predicted at 1-, 2-, and 3-year time points. For example, a 40-year-old unmarried BCLM patient was diagnosed with invasive lobular carcinoma (ILC), grade III, HER2 (-), ER ( +), PR ( +), with liver metastases, without bone or brain metastases, who underwent surgery and chemotherapy but did not receive neoadjuvant therapy, has a quantified score of 280 by the nomogram, and the predictive OS at 1-, 2-, and 3-year were 73%, 55%, and 44%, respectively.

**Table 2:** Univariate and multivariate Cox regression analyses of the training group

	**Univariate analysis**		**Multivariate analysis**		
**Variables**	**HR (95% CI)**	***P***	**HR (95% CI)**	***P***	
Age,years					
20–35	Reference		Reference		
35–59	1.119 (0.639–1.96)	0.693	1.173 (0.680–2.025)	0.564	
60–84	1.234 (0.809–2.176)	0.466	1.279 (0.736–2.222)	0.381	
85 +	2.393 (1.227–4.666)	0.010	2.362 (1.233–4.524)	< 0.01	
Tumor size,cm					
≤ 2 cm	Reference				
2.1-5 cm	0.849 (0.452–1.594)	0.611	—	—	
5.1-10 cm	1.584 (0.883–2.844)	0.122	—	—	
> 10 cm	1.217 (0.675–2.195)	0.513	—	—	
Race					
Black	Reference				
White	1.107 (0.875–1.402)	0.394	—	—	
Other	0.830 (0.606–1.137)	0.247	—	—	
Year of diagnosis			—	—	
2010–2011	Reference				
2012–2013	0.912 (0.737–1.128)	0.398	—	—	
2014–2015	0.868 (0.697–1.080)	0.205	—	—	
Laterality					
Left	Reference				
Right	0.996 (0.838–1.184)	0.966	—	—	
Primary site					
C50.0	Reference				
C50.1	0.924 (0.301–2.839)	0.891	—	—	
C50.2	1.118 (0.370–3.375)	0.842	—	—	
C50.3	1.651 (0.511–5.331)	0.401	—	—	
C50.4	1.316 (0.451–3.844)	0.615	—	—	
C50.5	1.401 (0.466–4.203)	0.547	—	—	
C50.6	0.331 (0.034–3.145)	0.336	—	—	
C50.8	1.165 (0.401–3.387)	0.779	—	—	
C50.9	1.272 (0.440–3.670)	0.656	—	—	
Histopathology					
Ductal	Reference		Reference		
Lobular	2.089 (1.293–3.375)	0.003	1.907 (1.204–3.022)	< 0.01	
Other	1.289 (1.008–1.648)	0.042	1.232 (0.972–1.562)	0.083	
Grade					
I ~ II	Reference		Reference		
III	1.547 (1.245–1.922)	< 0.01	1.607 (1.299–1.988)	< 0.01	
Unknown	1.528 (1.131–2.065)	0.005	1.473 (1.101–1.973)	< 0.01	
T stage					
T1	Reference				
T2	1.661 (0.829–3.326)	0.151	—	—	
T3	1.011 (0.522–1.957)	0.973	—	—	
T4	1.270 (0.681–2.365)	0.451	—	—	
N stage					
N0	Reference				
N1	1.187 (0.939–1.501)	0.151	—	—	
N2	1.258 (0.920–1.721)	0.149	—	—	
N3	1.182 (0.876–1.594)	0.272	—	—	
Marital status					
Unmarried	Reference		Reference		
Married	1.093 (0.895–1.334)	0.380	1.039 (0.857–1.259)	0.694	
Other	1.428 (1.098–1.857)	0.007	1.347 (1.046–1.734)	0.021	
Bone metastasis					
No	Reference		Reference		
Yes	1.564 (1.274–1.921)	< 0.01	1.551 (1.277–1.883)	< 0.01	
Unknown	2.535 (1.055–6.092)	0.037	2.899 (1.231–6.827)	0.014	
Brain metastasis					
No	Reference		Reference		
Yes	2.140 (1.607–2.848)	< 0.01	1.964 (1.521–2.535)	< 0.01	
Unknown	1.157 (0.708–1.889)	0.559	1.242 (0.787–1.962)	0.351	
Liver metastasis					
No	Reference		Reference		
Yes	1.517 (1.242–1.854)	< 0.01	1.560 (1.284–1.895)	< 0.01	
Unknown	0.772 (0.381–1.563)	0.472	0.732 (0.369–1.452)	0.372	
HER2					
Negative	Reference		Reference		
Positive	0.585 (0.468–0.731)	< 0.01	0.586 (0.474–0.726)	< 0.01	
Unknown	0.941 (0.597–1.483)	0.793	0.953 (0.608–1.491)	0.833	
ER					
Negative	Reference		Reference		
Positive	0.599 (0.458–0.785)	< 0.01	0.617 (0.473–0.804)	< 0.01	
PR					
Negative	Reference		Reference		
Positive	0.626 (0.483–0.812)	< 0.01	0.622 (0.484–0.801)	< 0.01	
Surgery					
No	Reference		Reference		
Yes	0.691 (0.539–0.883)	< 0.01	0.690 (0.546–0.871)	< 0.01	
Neoadjuvant therapy					
No	Reference		Reference		
Yes	0.647 (0.460–0.911)	0.01	0.651 (0.471–0.901)	< 0.01	
Chemotherapy					
No	Reference		Reference		
Yes	0.697 (0.568–0.856)	< 0.01	0.704 (0.576–0.862)	< 0.01	
Radiotherapy					
No	Reference				
Yes	0.949 (0.770–1.170)	0.625	—	—	

*CI* confidence interval, *HR* hazard ratio

### Nomogram validation

For the training group, the C-index of OS predicted by the nomogram (0.719, *p* < 0.01) was higher than the C-index predicted by AJCC 7th TNM stages (0.535, *p* < 0.01). Identically, C-index in testing group (0.695, *p* < 0.01) was also better than AJCC 7th TNM stages (0.506, *p* < 0.01). These results implied that the nomogram presented was more reliable than the AJCC 7th TNM stages for predicting OS in patients with BCLM.

**Fig. 2:**
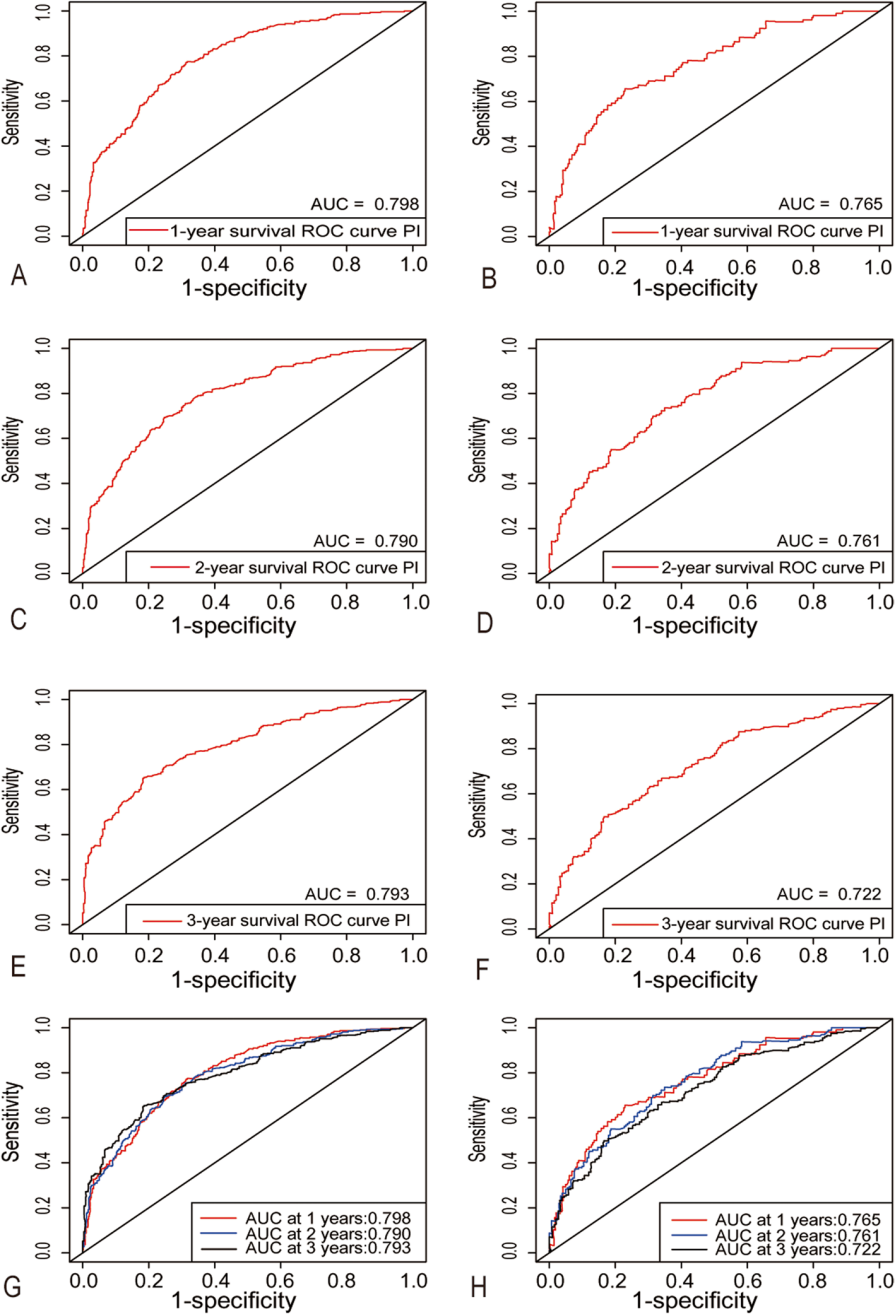
Time-dependent receiver operating characteristics curves of nomogram. 1-year survival in training group (**A**) and in testing group (**B**), 2-year survival in training group (**C**) and in testing group (**D**), 3-year survival in training group (**E**) and in testing group (**F**) and 1-,2-,3-year overall survival in training group (**G**) and in testing group (**H**)

Moreover, we calculated the AUC values by the area under the ROC curve (Fig. 2). In the training group, the AUC values for the nomograms to predict 1-, 2-, and 3-year OS were 0.798, 0.790, and 0.793, respectively. And in the testing group, the AUC values for the nomograms to predict 1-, 2-, and 3-year OS were 0.765, 0.761, and 0.722, respectively. The results demonstrated that the nomogram had an excellent predictive value in both training and testing groups at 1-, 2- and 3-year time points (Fig. 2). The calculation results of IDI and NRI were shown in Table 3, the usage of multiple variables to construct a comprehensive nomogram significantly improved the risk reclassification for 1-, 2-, and 3-year overall mortality prediction compared with the AJCC 7th staging system in both groups.

**Table 3:** The net reclassification improvement and integrated discrimination improvement of the nomogram

Category	NRI (95% CI)	*P*	IDI (95% CI)	*P*
Training group				
1-year OS	0.471 (0.337–0.555)	< 0.01	0.132 (0.103–0.177)	< 0.01
2-year OS	0.377 (0.271–0.463)	< 0.01	0.165 (0.127–0.219)	< 0.01
3-year OS	0.375 (0.293–0.453)	< 0.01	0.162 (0.127–0.209)	< 0.01
Testing group				
1-year OS	0.421 (0.299–0.581)	< 0.01	0.139 (0.101–0.217)	< 0.01
2-year OS	0.382 (0.273–0.498)	< 0.01	0.177 (0.129–0.244)	< 0.01
3-year OS	0.297 (0.206–0.395)	< 0.01	0.180 (0.126–0.259)	< 0.01

*CI* confidence interval, *IDI* integrated discrimination improvement, *NRI* net reclassification improvement, *OS* overall survival

**Fig. 3:**
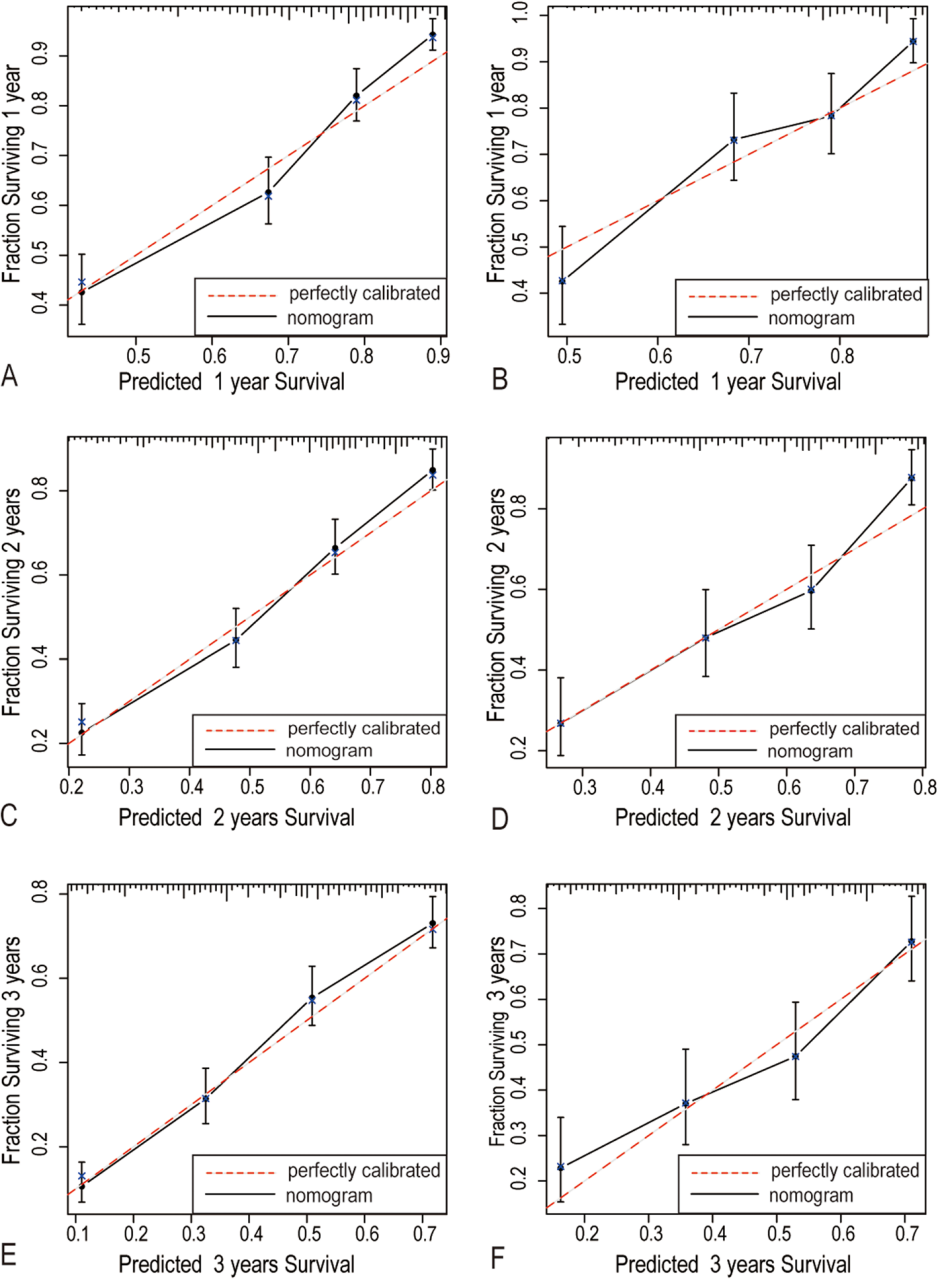
Calibration plots of 1-year (**A**-**B**), 2-year (**C**-**D**) and 3-year overall survival (**E**–**F**) in the training group and testing group

**Fig. 4:**
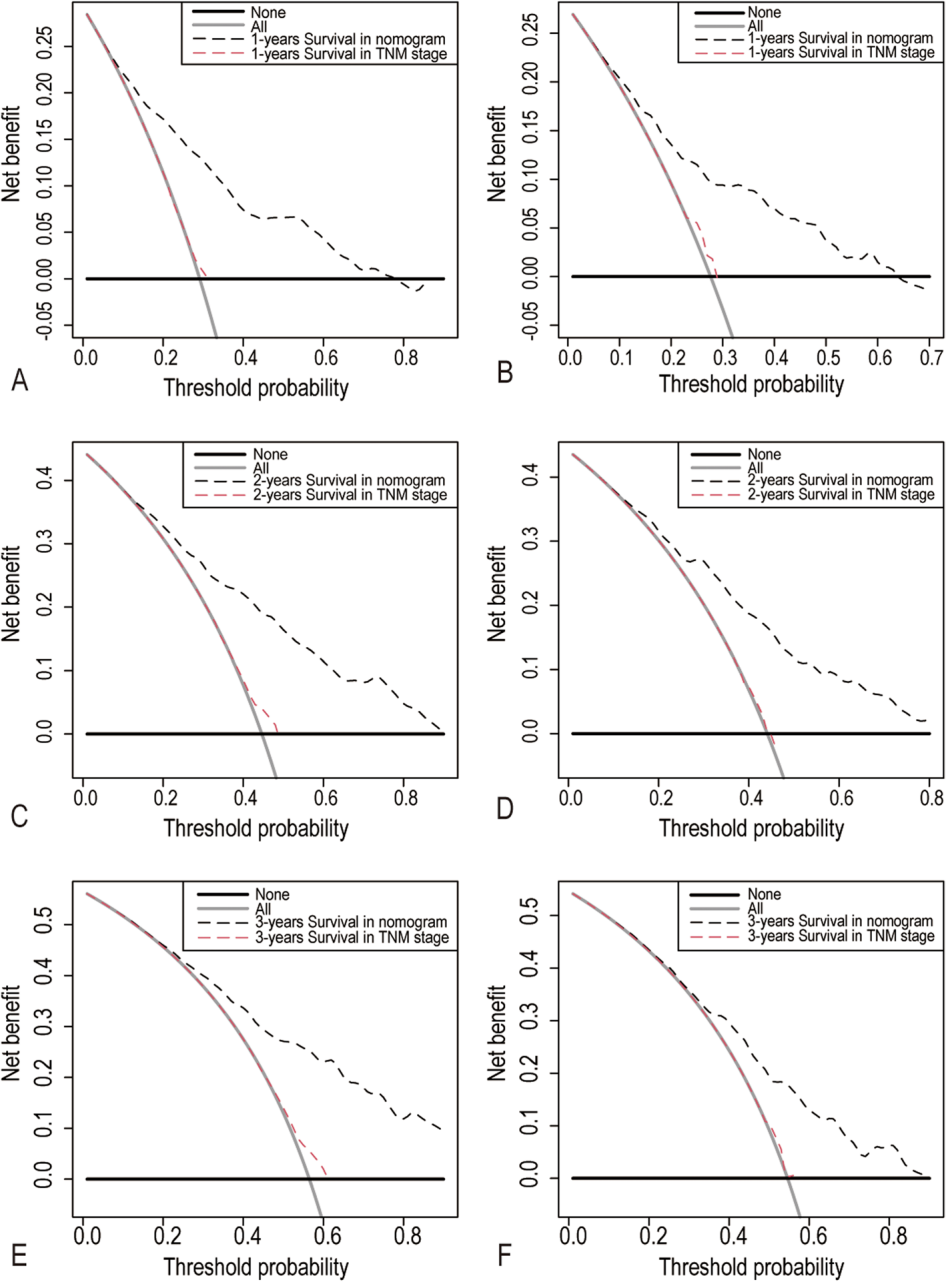
Decision curve analyses (DCA) of nomogram and TNM stage. 1-year (**A**-**B**), 2-year (**C**-**D**) and 3-year overall survival (**E**–**F**) in training group and testing group

The calibration plots have shown good agreement between observed and the nomogram predicted values in 1-, 2-, and 3-years of OS in the training group and testing group (Fig. 3). Compared with the AJCC 7th TNM stage, DCA showed that our nomogram had better net benefits at different threshold probabilities at different time points (Fig. 4). These results indicated that the nomogram we presented here were reliable with favorable clinical predictive value.

## Discussion

In this era of individualized therapy, accurate prediction of prognosis in patients with BCLM is very important for clinicians to make treatment options and communicate effectively with patients and family members. In the present study, we described sociodemographic and clinicopathological parameters in patients with lung metastases. In addition, we constructed variable factors related to the prognosis of lung metastases and verified the nomogram to predict the prognosis of BCLM patients.

Lung metastasis is one of the common site of metastatic breast cancer (MBC). Patients with lung metastases were all classified in stage IV based on AJCC TNM staging system, which could not supply individualized prognostic information for clinicians and patients. Therefore, there is an increasing need for developing effective models to predict the prognosis of BCLM patients. Previously, Xiao et al. proposed that the prognosis of patients with BCLM may be related to age, subtype, pathological grade, number of metastatic sites, and marital status. However, this study neither included the influence of treatment methods nor proposed a prognostic nomogram related to survival [18]. Here, treatment methods, including chemotherapy, surgery, and neoadjuvant therapy, were screened in our study, and a survival model was also proposed based on the relevant factors.

The nomogram was constructed by performing univariate and multivariate Cox regression analyses on the prognostic factors (age, race, marital status, years of diagnosis, laterality, primary site, tumor size, histopathology, grade, tumor stage, nodal stage, metastatic stage, clinical stage, and Immunohistochemical type) of the breast cancer database (ICD-0–3) in SEER. NRI and IDI suggested that our nomogram could better predict the OS of patients with BCLM compared with traditional TNM staging. The calibration curve was constructed and the results showed that the predictive performance of the nomogram at 1, 2, and 3 years was good, as the predicted OS probability and observed OS probability were in good agreement with the calibration curve (K = 1).

The nomogram can reflect the individual differences of patients because it quantifies the risk with specific values, which makes the nomogram better predicting OS than the traditional TNM staging. The accuracy of models was in direct proportion to their complexity, and we tried to make a balance between comprehensiveness and clinical usefulness. Therefore, we selected 13 prognostic factors with clinical importance and a small time-varying effect to construct a nomogram. In the present study, we declared several achievements of our findings. Firstly, our nomograms demonstrated that young patients (< 35) have a better prognosis, which is different from previous studies [19, 20]. It was probably related to the fact that young breast cancer patients have favorable treatment motivation, patient compliance, and chemotherapy tolerance [21]. Secondly, the OS of patients with lung metastasis of TNBC was worse than that of other types of BCLM [22]. Thirdly, in terms of pathological classification, our results showed that the prognosis of patients with invasive ductal carcinoma (IDC) was better than that of invasive lobular carcinoma (ILC). Fourthly, in terms of immunohistochemistry, patients with ER and PR had a better prognosis, presumably due to the opportunity for endocrine therapy. Finally, patients underwent neoadjuvant chemotherapy had a prolonged OS.

Chemotherapy and neoadjuvant therapy improve the prognosis of patients with BCLM [23]. Although some studies do not support neoadjuvant therapy has a positive effect on the long-term survival of MBC patients, our nomogram suggested that neoadjuvant therapy was beneficial for the survival of patients with BCLM, even if they developed lung metastases after neoadjuvant therapy [24]. It may be because neoadjuvant therapy can help clinicians to choose more tailored therapy based on neoadjuvant response.

Social, mental, and emotional stress are thought to be associated with cancer. As a systemic disease, breast cancer might be the result of a complex interaction of physiological and psychosocial factors. In our study, a sociodemographic factor- marital status was included in our research. Accumulating evidence showed that marital status was an independent prognostic factor affecting the survival of breast cancer patients [25]. Here, we found that the prognosis was worse when a patient was in a relationship status of "divorced" or "widowed" than “married” or “unmarried”. It was speculated that in addition to the physical burden of the tumor, these patients had to face the emotional stress and grief of losing their spouse and the material support from their partners. Some studies suggested that the frequency of “widows” receiving chemotherapy and their tolerance of chemotherapy were lower than those of “married” patients, which might be related to the inhibited response of peripheral blood lymphocyte stimulation of widowed patients, which led to worse OS of widowed patients than those of other marital status [26, 27].

Our study, based on SEER database, improved the accuracy of independent risk factors that predict prognosis in patients with BCLM. However, the data of patients with BCLM we chosen was recorded from 2010 to 2015, which brought limitations to this study. In our opinion, the number and maximum diameter of lung metastases, Ki67 expression level, ER expression intensity, lifestyle, economic conditions, and other factors might be associated with the prognosis. Unfortunately, these information could not be obtained from the 2010–2015 SEER database. Therefore, other relevant factors are needed to further correct and supplement the nomogram in future studies.

## Data Availability

The SEER database we used in the present stady is publicly accessible (https://seer.cancer.gov/data/access.html).
